# The Chemical Composition and Health-Promoting Benefits of Vegetable Oils—A Review

**DOI:** 10.3390/molecules28176393

**Published:** 2023-09-01

**Authors:** Mingke Tian, Yuchen Bai, Hongyu Tian, Xuebing Zhao

**Affiliations:** 1 Beijing Key Laboratory of Flavor Chemistry, Beijing Technology and Business University, Beijing 100048, China; 2 Key Laboratory of Industrial Biocatalysis, Ministry of Education, Tsinghua University, Beijing 100084, China; zhaoxb@mail.tsinghua.edu.cn; 3 Institute of Applied Chemistry, Department of Chemical Engineering, Tsinghua University, Beijing 100084, China

**Keywords:** vegetable oil, chemical compositions, health effect, nutritional properties, functional ingredients, edible oils, diseases prevention

## Abstract

With population and economic development increasing worldwide, the public is increasingly concerned with the health benefits and nutritional properties of vegetable oils (VOs). In this review, the chemical composition and health-promoting benefits of 39 kinds of VOs were selected and summarized using Web of Science ^TM^ as the main bibliographic databases. The characteristic chemical compositions were analyzed from fatty acid composition, tocols, phytosterols, squalene, carotenoids, phenolics, and phospholipids. Health benefits including antioxidant activity, prevention of cardiovascular disease (CVD), anti-inflammatory, anti-obesity, anti-cancer, diabetes treatment, and kidney and liver protection were examined according to the key components in representative VOs. Every type of vegetable oil has shown its own unique chemical composition with significant variation in each key component and thereby illustrated their own specific advantages and health effects. Therefore, different types of VOs can be selected to meet individual needs accordingly. For example, to prevent CVD, more unsaturated fatty acids and phytosterols should be supplied by consuming pomegranate seed oil, flaxseed oil, or rice bran oil, while coconut oil or perilla seed oil have higher contents of total phenolics and might be better choices for diabetics. Several oils such as olive oil, corn oil, cress oil, and rice bran oil were recommended for their abundant nutritional ingredients, but the intake of only one type of vegetable oil might have drawbacks. This review increases the comprehensive understanding of the correlation between health effects and the characteristic composition of VOs, and provides future trends towards their utilization for the general public’s nutrition, balanced diet, and as a reference for disease prevention. Nevertheless, some VOs are in the early stages of research and lack enough reliable data and long-term or large consumption information of the effect on the human body, therefore further investigations will be needed for their health benefits.

## 1. Introduction

Vegetable oil is an indispensable ingredient in the human daily life diet, not only due to its sensory attributes when used as a cooking medium, but also as an supply energy to maintain body normal temperature [1]. VOs provide essential fatty acids (EFAs) which serve as carriers for liposoluble vitamins, act as precursors for steroid hormones and prostaglandins synthesis [2], and play a significant role in health protection and disease prevention. The consumption of edible vegetable oils (EVOs) has been rising steadily due to urbanization, per capita income rise, and shifts to obesogenic diets around the world [3].

In terms of production and consumption, palm oil, soybean oil, and rapeseed oil are the three main types of vegetable oil that have dominated the world market due to their high yield, high oil production rate, and good stability. It was reported that the production of soybean accounts for 60% of the world’s total edible oil production [4]. Additionally, palm oil accounts for 30% of the total production and its consumption has increased rapidly in the past several decades, particularly in India and China [5]. Differently, soybean oil, rapeseed oil, palm oil, and peanut oil share the greatest consumption market in China. There is a high demand for vegetable oil worldwide, with global consumption in 2022 at 212.82 million tons, an increase of 1.25 times compared to 2014, while its production, import, and export quantities also basically maintain a steady growth trend [6]. In recent years, due to limited arable land, the difficulty of expanding oilseed crop production, and the impact of COVID-19, the refined production of EVOs in China decreased to 48.819 million tons in 2022, with a decrease of 912,000 tons over the previous year. At the same time, with the rapid growth of consumption, the self-sufficiency of EVOs has been decreasing year by year, resulting in a high dependence on the external market [7]. The growth of population, the improvement of living standards, long life span, and the prevalence of chronic diseases would further break the balance of supply and demand of the consumption of EVOs. Ensuring the supplement of EVOs is of great significance for social stability.

In recent years, it has been found that only several kinds of the VOs are used by people for cooking, while minor VOs with relatively small market shares and production volumes are relatively underutilized compared to major vegetable oils, such as rice bran oil, safflower oil, and almond oil [8]. Although these new types of VOs are often expensive and not to the taste preferences of people in different regions, their special nutritional characteristics and variety have attracted more attention due to the increasing health conscious of the public [9]. In addition, scholars have found that the extracted seed oils from some typical popular fruits and vegetables, such as avocado, jackfruit, papaya, and pumpkin, can provide extra potential health effects to the human body. For instance, pumpkin seed oil is rich in tocopherols, unsaturated fatty acids, and phytosterols, exhibiting an excellent antioxidant activity [10]. 

Therefore, this review summarizes the recent research progress on the chemical composition of 39 kinds of VOs including soybean oil, rapeseed oil, and palm oil, etc., which are generally consumed by people, and various kinds of minor VOs, perilla seed oil, evening primrose oil, and grape seed oil, etc. The chemical composition, including fatty acids, tocols, phytosterols, squalene, carotenoids, phenolics and phospholipids, was summarized and discussed. In particular, the prompting health benefits of VOs intake according to each functional substance are discussed. Antioxidant activity, prevention of CVD, anti-inflammatory, anti-obesity, anti-cancer, diabetes treatment, kidney and liver protection are analyzed in relation to certain functional substance in VOs, providing recommendations for general public nutrition, balanced diet, and disease prevention. To achieve a comprehensive review on chemical composition of VOs and their health-promoting benefits, research and review articles were searched and selected using Web of Science ^TM^ as the main bibliographic database covering the years from 2007 to 2023 using the key words of “vegetable oil”, “edible oil”, by which 5416 articles were retrieved. Then, refining by the “health benefits” or “health effect”, and considering the titles, relevance and subject areas, 141 articles were selected for summarization and analysis.

## 2. Chemical Composition

### 2.1. Fatty Acid Composition

Fatty acids (FAs) are crucial as the main components of biological matter in humans and organisms. Based on the saturated or unsaturated state hydrocarbon chains, fatty acids can be categorized into several groups, including saturated fatty acid (SFA), monounsaturated fatty acid (MUFA), and polyunsaturated fatty acid (PUFA). In most MUFAs, the position of the double bond is almost always between C9 and C10 (∆^9^). EFAs are PUFAs that cannot be synthesized by the human body and must be provided by food. It derives two “families” of ω-3 (n-3) and ω-6 (n-6) FAs. Linoleic acid (LA) is metabolized to the long-chain ω-6 polyunsaturated arachidonic acid (AA), and *α*-linolenic acid (ALA) can be metabolized to the long-chain ω-3 polyunsaturated eicosatetraenoic acid (EPA) and docosahexaenoic acid (DHA) [11].

In VOs, FAs have an even number of C-atoms in *cis* configuration and vary widely in composition and content, but typically, one type of fatty acid will predominate. For example, the major fatty acid found in olive oil is oleic acid (OA), which is classified as “monounsaturated oil”, whereas the predominant fatty acid in sunflower oil is LA, and in flaxseed oil is ALA, therefore, they are often referred to as “polyunsaturated oil”. An exception is coconut oil, which is an almost saturated oil. In addition, most culinary oils used at home tend to be predominantly unsaturated.

The fatty acid composition of VOs is shown in Table A1. The top three VOs with SFA content were coconut oil (92.10%), jackfruit seed oil (49.13%), and palm oil (44.15%). Among the SAFs, lauric acid is the highest ranging from 47.70% to 49.57%, while PUFA is the lowest at 1.60–1.90%, which makes coconut oil unique [12]. Pomegranate seed oil contains the smallest amount of SFAs at only 5.35% [13]. Regarding MUFAs, almond oil has the highest MUFA content at 77.07%, followed by olive oil (76.62%), and papaya seed oil (76.10%) [14,15]. In contrast, jackfruit seed oil has the lowest MUFA content (4.15%). Some VOs with high PUFA content are pomegranate seed oil (87.87%), evening primrose oil (83.40%), safflower oil (79.10%), and flaxseed oil (76.94%). Naturally, they are referred to as “polyunsaturated oil”. The major fatty acids available in oils were palmitic acid (C16:0), OA(C18:1), LA (C18:2), and linolenic acid (C18:3). The LA (C18:2) content was the highest (79.00%) in safflower oil [16]. Linolenic acid has two isomers: ALA (C18:3n-3)) and *γ*-linolenic acid (GLA)(C18:3n-6), the former is more common, and the latter is only found in a small number of VOs, such as in evening primrose oil as GLA at 9.50%, whereas as ALA it is only 0.10% [17]. Flaxseed oil, perilla seed oil, and *Eucommia ulmoides Oliver* seed oil contained high amounts of ALA, which were, 61.06%, 51.87%, and 59.87%, respectively [15].

### 2.2. Tocols

Tocols refer to tocopherols and tocotrienols with vitamin E activity. Tocopherols are primarily found in VOs and act as antioxidants to prevent the peroxidation of unsaturated lipids via Chain-breaking reactions. They are composed of a polar moiety derived from tyrosine, the chromanol ring, and a hydrophobic phytol derived side chain. Tocopherols consist of *α-, β-, γ,* and *δ*-tocopherols, with *α*-tocopherol having the highest physiological activity, but *δ*-tocopherol has the strongest antioxidant effect. Among them, tocopherols and tocotrienols, especially *α-* and *γ*-tocopherol, are the most effective lipid-soluble antioxidants [18]. As presented in Table A2, pomegranate seed oil was found to have the highest level of total tocols (5246.0 mg/kg), followed by soybean oil (1774.6 mg/kg) and cress oil (1754.0 mg/kg), while palm kernel oil had the lowest concentration of only 34.0 mg/kg, as well as coconut oil which also had a low concentration (35.0 mg/kg) [19]. Sunflower oil was identified as the best source of *α*-tocopherol (570.5 mg/kg), with safflower oil (451.2 mg/kg), cottonseed oil (386.2 mg/kg), and camellia oil (378.0 mg/kg) in succession [20]. There were four oils with the highest contents of *γ*-tocopherol: pomegranate seed oil (3829.0 mg/kg), soybean oil (1639.7 mg/kg), cress oil (1550.0 mg/kg), and *Eucommia ulmoides Oliver* seed oil (906.2 mg/kg) [20]. *δ*-tocopherol was not found in evening primrose oil, palm oil, or *Torreya grandis* seed oil, while it was highest in soybean oil (304.7 mg/kg). *α*- and *γ*-tocopherols are the main types of tocopherols found in almost all vegetable oil, the exception being *Torreya grandis* seed oil, where *β*-tocopherol accounts for 89% of its tocopherols [21]. The *α*-tocopherol in safflower oil accounted for 93.0% of the tocols, which was the highest among VOs. Sesame and peony seed predominantly contain 95.0% and 92.7% *γ*-tocopherol [22]. A unique compound in flaxseed oil is plastochromanol-8, a *γ*-tocopherol with a side chain twice as long as *γ*-tocopherol which is the most potent antioxidant of all tocopherols [23]. Interestingly, some VOs contain higher levels of tocotrienols than tocopherols, for example, grape seed oil has *α*-tocotrienol and *γ*-tocotrienol as its most abundant tocols constituents; in rice bran oil *γ*-tocopherol is the most important tocols, with a content of 823.4 mg/kg; in palm oil, tocotrienols represent 75.0% to 79.0% of the total tocols [20,24].

### 2.3. Phytosterol

Phytosterols are widespread in plants and are similar to cholesterol in terms of chemical structure and physiological functions. Phytosterols belongs to the triterpene family with a tetracyclic ring and a side chain linked to carbon 17, they are essential biomolecules for human health and must be taken from food [25]. In higher plants, phytosterols are primarily synthesized from mevalonic acid (MVA) through the mevalonate pathway that produces terpenes and steroids [26]. In lower plants, an alternative pathway goes through 2-c-methyl-d-erythritol 4-phosphate instead of MVA [27]. 

Actually, more than 100 different phytosterols have been identified in VOs, among them *β*-sitosterol, campesterol, stigmasterol, brassicasterol, and ∆5-avenasterol were the major types. As shown in Table A3, *β*-sitosterol was the main phytosterol in VOs, and the highest amount of *β*-sitosterol was presented in rice bran oil (735.17 mg/100 g), followed by corn oil (661.70 mg/100 g), sour cherry seed oil (601.83 mg/100 g), and sesame oil (555.00 mg/100 g). The total phytosterol content was ordered slightly different from the *β*-sitosterol content, and was rice bran oil (1891.82 mg/100 g), corn oil (1032.07 mg/100 g), rapeseed oil (893.84 mg/100 g), and flaxseed oil (775.20 mg/100 g) in descending order [28]. Not only is there a high content of *β*-sitosterol in corn oil, but campesterol also accounts for a significant portion, which can be as high as 197.32 mg/100 g. Referring to the content of campesterol, rapeseed oil has the highest content (288.59 mg/100 g) among all VOs, and also contains much higher levels of brassicasterol than others, ranging from 51 to 136 mg/100 g. Most of the VOs are dominated by *β*-sitosterol; however, in camellia oil, the phytosterols are dominated by sitostanol which accounts for 65% of the total sterols [29]. It is worth noting that rice bran oil occupies several first places, including campestanol (221.20 mg/100 g), stigmasterol (132.90 mg/100 g), *β*-sitosterol (735.17 mg/100 g), ∆5-Avenasterol (157.41 mg/100 g), cycloartenol (31.08 mg/100 g), cycloartenol (156.25 mg/100 g) and 24-methylene-cycloartanol (222.88 mg/100 g), which means it is rich in almost all types of phytosterols [28].

### 2.4. Squalene

Squalene is a natural terpenoid hydrocarbon containing six isoprene units and is an intermediate product in the biosynthesis of phytosterols. It was initially isolated from deep-sea shark liver, hence the name. It is one of the most biologically active constituents and is an important unsaponifiable component of VOs, it is biosynthesized at the initial steps of the mevalonate pathway in the body as well as through dietary intake of polyunsaturated hydrocarbons [9]. 

The amount of squalene varies in different types of VOs (Table 1). In general, the content of squalene in vegetable oil is generally 2–300 mg/100 g. Olive oil had the highest level of squalene, especially virgin olive oil, squalene content up to 153.4–747.4 mg/100 g [30]. Apart from olive oil, other important plant sources of squalene include rice bran oil (318.9 mg/100 g), corn oil (256.8 mg/100 g) and pumpkin seed oil (198.0 mg/100 g) [31,32]. In crude sunflower oil, squalene content is typically between 15.0 and 20.0 mg/100 g, though higher levels of up to 27.1 mg/ 100 g have been reported [22], whereas evening primrose oil was found to be free from squalene. From the table, it can be seen that there is a significant difference in the content of squalene within the same VOs reported in the literature, which may be caused by different factors such as gene type, growth conditions, climate, and processing methods.

### 2.5. Carotenoids

Carotenoids are fat-soluble pigments produced by plants and algae as well as several species of bacteria and fungi. They are the most common pigments in VOs. They belong to the tetraterpene group and are produced from eight isoprene molecules containing 40 carbon atoms. They are classified into two types: xanthophylls, which contain oxygen, such as lutein and zeaxanthin; and carotenes, which are purely hydrocarbons without oxygen, such as *α*-carotene, *β*-carotene, cryptoxanthin, and lycopene [22].

There is a range of carotenoids content in VOs. The highest content of carotenoids was found in tomato seed oil (765.7 mg/kg), followed by rapeseed oil (358.7 mg/kg), flaxseed oil (76.9 mg/kg), sea buckthorn seed oil (55.3 mg/kg), and olive oil (44.8 mg/kg), whereas rice bran oil was found to be free of carotenoids [9]. Carotenoids are known as a vitamin A precursor and have vitamin A activity. Tomato seed oil and rapeseed oil have a high content of total carotenoids, so they are regarded as good sources of vitamin A. Tomato seed oil (765.7 mg/kg), flaxseed oil (76.9 mg/kg), sea buckthorn seed oil (55.3 mg/kg), and olive oil (50.3 mg/kg) had relatively high *β*-carotene content, which is generally a predominant carotenoid in VOs [23]. For lutein, rapeseed oil contains up to 95 mg/kg, which is higher than sunflower oil (12.4 mg/kg) and flaxseed oil (11.6 mg/kg), and is a good source of vitamin A [34]. The carotenoid contents of representative VOs were summarized in Table 2.

### 2.6. Total Phenolics

Phenolic compounds and phenolic acids are a group of molecules that contain at least one aromatic ring and one hydroxyl group, which are important secondary metabolites present in almost all VOs [37]. Phenolic compounds can be classified into phenolic acids, flavonoids, stilbenes, and lignans [38]. 

Phenolic compounds are important for the oxidative stability of PUFAs in VOs [39]. The total free content of phenolic compounds and the phenol profile in VOs are very diverse and depend on the source of oil and production method. Due to the plant source agriculture treatment and technology of oil extraction, various phenolic compounds were identified, such as acids, phenolic alcohols, flavonoids, secoiridoids, lignans, and their metabolic derivatives. 

Table 3 provided the total phenolic contents of representative VOs. There was a great difference in the level of total phenolics among different VOs, ranging from 0.4 mg/kg to 59,300.0 mg/kg depending on the source oil and production method. Coconut oil (59,300.0 mg/kg), *Torreya grandis* seed oil (12,630.0 mg/kg), perilla seed oil (11,090.0 mg/kg), rice bran oil (10,220.0 mg/kg), and pomegranate seed oil (9000.0 mg/kg) showed a much higher total phenolic content than other oils [40]. The wide span of phenolic concentration in VOs is the result of a range of production factors, including the diversity of seeds, regions, agricultural techniques, the maturity of the harvest, the methods of extraction, processing, and storage of the VOs [41]. The types of phenolic compounds in VOs are diverse. The main phenolic compounds in rapeseed oil are 4-vinylsyringol (canolol), accounting for 85% of total phenolics, while ferulic and p-coumaric acids occurred in smaller amounts [42]. However, 24 phenolic compounds including benzoic acid, cinnamic acid, hydroxyphenyl acetic acid and flavan-3-ol, flavonols, flavones, and dihydroflavones were found in camellia oil, of which 13 phenolic compounds were reported for the first time [43].

The phenolic acids and their derivatives accounted for a small part of the total polyphenols, conversely, they evidently affect human health. A review of the literature data has shown that phenolic acid content in pumpkin seed oil is as high as 164.800 mg/kg, followed by grape seed oil (92.490 mg/kg), camellia oil (31.243 mg/kg) and corn oil (8.850 mg/kg), whereas the phenolic acid content of rice bran oils is only 0.004 mg/kg [45]. The major phenolic acids and their derivatives of flaxseed oil are p-hydroxybenzoic, ferulic acids, vanillic acid, and vanillin. In olive oil, vanillic, protocatechuic, caffeic and p-coumaric prevailed, accounted for 59.5%, 22.3%, 12.2% and 6.0% of all phenolic acids, respectively.

### 2.7. Phospholipids

Phospholipids are a large class of lipid molecules which contain phosphate groups, hydrophilic heads, and lipophilic tails. VOs usually contain between 0.1% and 3.0% phospholipids [11]. According to their characteristic groups, phospholipids are mainly categorized into phosphatidylcholine (PC), phosphatidylethanolamine (PE), phosphatidylglycerol (PG), phosphatidylinositol (PI), phosphatidylserine (PS), phosphatidic acid (PA), lyso-phosphatidylcholine (LPC), and their acyl forms. 

Phospholipids appear in the form of emulsifiers and nutritional supplements, which were found to be important components in flaxseed oil. They can facilitate the dispersion of solid particles in the aqueous phase and improve the multiphase texture, thus showing various beneficial processing properties and physiological effects. The phospholipid components in flaxseed oil included PE (27–40%), PI (29–32%), PC (7–18%), LPC (8–21%), PG (1–4%), and PA (1–9%) [15]. Palmitic (C16:0), stearic (C18:0), oleic (C18:1), linoleic (C18:2), and linolenic acid (C18:3) were the main fatty acyl chain of phospholipids [48].

In addition to the above active compounds, some special nutrients also exist in VOs, such as green tea polyphenols in camellia oil, *γ*-oryzanol in rice bran oil, lignans in flaxseed oil, resveratrol in peanut oil, and lycopene in tomato seed oil [49]. These nutrients may significantly contribute to the biological activities of VOs.

## 3. Health-promoting Benefits of Vegetable Oil

The composition of vegetable oil is known to include not only EFAs, but also micronutrients such as phytosterols, tocopherol, carotenoid, and phenolics, etc. The functional value of VOs includes their reduction in free radicals, lowering of blood cholesterol, and prevention chronic diseases. For these effects, which result from the functional compounds in VOs, the health-promoting benefits are discussed in the following sections. 

### 3.1. Antioxidant Activity

Reactive oxygen species (ROS) and free radicals (FR) are generated during metabolism in vivo as well as during exposure to adverse pathophysiological conditions [50]. ROS or FR can initiate oxidative stress, wherein the single electron seeks to pair with biological macromolecules such as lipids, proteins, and DNA, leading to damage along with lipid peroxidation. The generation of ROS contributes to many chronic diseases such as atherosclerosis, diabetes, inflammatory diseases, and cancers. Therefore, some active components, such as tocopherols, phytosterols, carotenoids, and phenolics, which have free radical scavenging capacity in VOs, may combat the cell oxidative damage and benefit human health. The presence of hydroxyl groups (-OH) in phenolics, tocopherols, and phytosterols may help to scavenge FR and prevent oxidation [51]. The antioxidant ability of functional components, their correlation to VOs, and the reaction mechanisms are summarized in Table 4.

The antioxidant mechanism of tocopherols is to provide electrons to FR making them inactive compounds through which they can be exchanged to tocopherol FR. Tocopherol radicals are stable and can form dimers, trimers, or be converted to tocopherol quinine, which is metabolized and excreted [10]. Recent studies have shown that the high levels of *γ*-tocopherol in pomegranate seed oil contribute to its powerful antioxidant capacity [54]. Cress oil is a relatively stable oil due to the presence of a high concentration of antioxidants such as tocopherols and carotenoids [55]. Soybean oil also exhibits excellent antioxidant properties, which are associated with tocopherols, suggesting that it can effectively quench FR, and reduce lipid peroxidation [56].

Phytosterols have antioxidant potential due to their ability to inhibit oxidative degradation of lipids and their strong scavenging effect on 1,1-diphenyl-2-picrylhydrazyl (DPPH) radicals and hydroxyl radicals. Rice bran oil, which is rich in unsaturated linoleic and oleic acids as well as bioactive compounds such as *γ*-oryzanol, phytosterols, tocopherols, and tocotrienols, exhibited significant antioxidant activity in the inhibition of cholesterol oxidation. The higher levels of stigmasterol and *β*-sitosterol in corn oil give it a higher scavenging capacity for DPPH FR than grape seed and coconut oils, thus exhibiting its antioxidant activity [57].

Carotenoids are essential antioxidants that are able to donate an electron and neutralize FR, resulting in suppression of the excess FR production, which inhibits the deterioration of internal redox balance and terminates some chain reactions [58]. Carotenoids have been well-characterized for their antioxidant activity in vitro, and recent data have also shown their ability to specifically limit PUFA peroxidation in lipid membranes [59]. Current research has identified a number of types of seed oils from new sources that contain high levels of antioxidant compounds and have the potential to be incorporated into foods as functional edible oils or as natural antioxidants. Tomato seed oil has been shown to contain high levels of carotenoids, particularly *β*-carotene, which has high antioxidant activity due to its ability to burst singlet oxygen and trap peroxyl radicals [60]. In addition, sea buckthorn seed oil is high in carotenoids and is a potent antioxidant within in vitro model systems [61].

The mechanism of phenolic compounds is to prevent the generation of FR in the body and block the oxidation reaction of PUFAs or low-density lipoproteins (LDLs) induced by FR. Coconut oil, especially virgin coconut oil, has the highest total phenolic content among the 39 VOs, as well as has high levels of polyphenols, tocopherols, phytosterols, and monoglycerides, resulting in excellent free radical scavenging activity and high antioxidant capacity [62]. Additionally, a study on *Torreya grandis* seed oil showed that its samples exhibited concentration-dependent antioxidant activity through a DPPH free radical scavenging test and a *β*-carotene bleaching test to assess lipid peroxidation inhibitory activity, leading to the conclusion that the high total phenolic compound content may be attributed to its potent antioxidant activity [63].

Furthermore, EFAs, especially ALA, are crucial to antioxidant capacity. The antioxidant, antihyperglycemic, and antihyperlipidemic activity of flaxseed oil can be attributed to the presence of linolenic acid and its metabolites EPA and DHA [64]. After ingestion of LA, the activity of glutathione peroxidase (GSH-Px) and superoxide dismutase (SOD) is enhanced, and the generation of free radical metabolite malondialdehyde (MDA) decreases, thus achieving free radical scavenging, reduced cell damage, and improved tissue and organ function. Similarly, the activity of GSH-Px and SOD in the liver and kidney of mice was increased by grape seed oil, and the MDA content in all organs was decreased. Millan-Linares et al. reported that due to the high content of LA (58–78%), the unsaponifiable fraction isolated from grape seed oil attenuated oxidative and inflammatory responses in lipopolysaccharide (LPS)-treated human primary monocytes by suppressing intracellular production of ROS and nitrite levels as a consequence of reduced nitric oxide synthase-2 (Nos2) gene expression [65].

### 3.2. Prevention of Cardiovascular Disease (CVD)

CVD is the number one leading cause of death worldwide, causing an estimated 17.9 million deaths each year [66]. The progress of inflammation in the blood vessels and endothelial dysfunction causes an atherosclerotic lesion in the arteries, which further induces stroke and myocardial dyslipidemia, as is indicated by elevated concentrations of total cholesterol (TC), low-density lipoprotein cholesterol (LDL-C), and triglyceride (TG), as well as low concentrations of high-density lipoprotein cholesterol (HDL-C) which continues to be a major CVD risk factor [67]. Additionally, age, smoking, detrimental diet, lack of exercise, psychological stress, diabetes, and obesity increase this burden. The studies summarized in Table 5 show the consumption of representative nutritionally rich VOs potentially reduces the risk of CVD, and attributed this to the content of MUFA, PUFA, and phytosterols. The substitution of SFAs, such as animal fats and dairy products, with VOs that are high in MUFA and PUFA is emphasized as regulating the blood lipid profile.

MUFA helps to prevent the oxidation of LDL-C. LDL particles which undergo oxidation in the arterial wall result in the atherosclerotic process. The Mediterranean diet which is rich in MUFA has been proven to decrease cardiovascular morbidity and mortality [71]. Oleic acid (OA) can decrease plasma triacylglycerol and cholesterol concentrations without affecting plasma HDL-C levels in healthy normolipidemic subjects [68]. A recent meta-analysis focusing on the effects of different dietary sources of MUFAs on CVD provided evidence that olive oil was associated with a significant risk reduction in all-cause mortality, cardiovascular events, and stroke [72]. Almond oil dominated by OA has been proven to improve endothelial function effectively. If endothelial function is damaged, it will lead to atherosclerotic vascular disease. Therefore, almond oil is beneficial to cardiovascular system [73]. 

PUFA, especially linoleic acid (LA) and linolenic acid, in vegetable oil helps to regulate the blood plasma TG levels in patients with dyslipidemia, lower the blood pressure, and protect against coronary heart disease [74]. Marangoni et al. reported that epidemiological studies have shown that adequate intake of LA reduces plasma LDL-C and illustrated that dietary intervention studies have shown that replacing 5% of dietary SFA energy with ω-6 PUFAs reduces LDL-C by up to 10%, the risk of coronary events by 13%, and the risk of coronary death by 26% [75]. Several studies have shown that PUFA protects the blood vessels and heart by regulating membrane phospholipids, thereby improving cardiac mitochondrial function and energy production and reducing TG concentrations [76]. Therefore, VOs rich in PUFA are widely recommended as a way to reduce the risk of CVD. Han et al. reported that flaxseed oil is rich in ω-3 PUFAs, and that ALA (39.00–60.42% of the major fatty acid) suppresses the biosynthetic pathway of cholesterol and TG by regulating the expression of sterol regulatory element-binding proteins 3-hydroxy-3-methylglutaryl coenzyme-A (HMG-CoA) reductase, sterol regulatory element-binding proteins 1c (SREBP-1c), and acetyl-CoA carboxylase, thus illustrating that partial replacement of lard with flaxseed oil can significantly alleviate atherosclerosis symptoms, improve oxidative stress, reduce lipid and inflammatory abnormalities, and promote cardiovascular health [77,78]. Several studies have also shown that perilla seed oil, sea buckthorn seed oil, walnut oil, and other polyenoic VOs have hypolipidemic health effects and can prevent and treat CVD due to the high content of LA and linolenic acid which reduce the plasma concentrations of TC, LDL-C, very low density lipoprotein cholesterol (VLDL), apolipoprotein B, and apolipoprotein A-1 [79]. Pomegranate seed oil is rich in PUFAs, and a recent study using a hamster model has shown that it is effective in lowering plasma and liver cholesterol, as well as in increasing HDL/LDL ratios when partially replacing saturated fat in a high-fat diet [80].

Phytosterols are well known for their cholesterol-lowering ability. An average daily dose of 2 g phytosterols lowers plasma LDL-C by approximately 0.31–0.34 mmol/L or 8–10% within 3–4 weeks [81]. Their ability to lower LDL-C concentrations through reduced intestinal absorption has been well documented [67]. Some bioactive accompaniments of grape seed oil, such as phytosterols and polyphenols, have also been shown to prevent and improve hypertension [82]. In addition, the high phenolics, phytosterols, and tocopherols in cold-pressed rapeseed oil help to reduce the absorption of dietary cholesterol and decrease the risk of hyperlipidemia and CVD. Thus, cold-pressed rapeseed oil can be used as a supplement to offer a cholesterol-lowering effect [35]. Yang and coworkers estimated phytosterol intake in the Chinese diet, and the results show that the phytosterol contents of rice bran oil, corn oil, and rapeseed oil are higher than those of other VOs and play a vital role in the reduction in cholesterol in blood, as well as decreasing cardiovascular morbidity [28]. Additionally, phytosterols which are present in high concentrations in pomegranate seed oil inhibit the absorption of intestinal cholesterol. Therefore, they play a natural preventive role in CVD. In 2011, the European Food Safety Authority (EFSA) approved a claim that extra virgin olive oil (EVOO) polyphenols protect blood lipids against oxidative stress at a minimal dose of 5 mg/kg/day [83].

### 3.3. Anti-Inflammatory

Inflammation is one of the primary factors associated with the causation and progression of several lifestyle disorders, it is associated with many diseases, including obesity, type 2 diabetes mellitus (T2DM), cancer, anaphylaxis, arthritis, and non-alcoholic fatty liver disease (NAFLD), etc. Additionally, inflammation can cause genetic defects, disrupt immune regulation, and damage the body tissues [84]. Diet and inflammatory response are recognized as strictly related, and PUFAs present in vegetable oil, particularly ω-3 polyenoic acids, can achieve anti-inflammatory effects by competing with AA for metabolism and participating in the regression of inflammation and tissue repair, thus affecting the nuclear factor-*κ*B (NF-*κ*B) pathway and altering the lipid raft pathway, among other mechanisms. LA can reduce the levels of inflammation-related factors such as interleukin-1*β* (IL-1*β*), tumor necrosis factor alpha (TNF-*α*), and nitric oxide (NO), and has a significant ameliorating effect on both acute and chronic inflammation [79]; ALA and GLA possess anti-inflammatory and anti-allergic properties to improve the immune system, as well as serve as a precursor of the prostaglandin and tissue hormones. The functions of some VOs as anti-inflammatories and their mechanisms are summarized in Table 6.

A large body of evidence has demonstrated the efficacy of ω-3 long-chain polyunsaturated fatty acids (ω-3 LCPUFA) in NAFLD conditions, due to their anti-inflammatory, immunomodulatory, and anti-viral properties. Therefore, intervention with ω-3 LCPUFA, an effective pharmaconutrient, along with the standard treatment for COVID-19 may be useful in the management of NAFLD spectrum in COVID-19 patients with pre-existing NAFLD conditions by resolving the inflammatory cytokine storm, thereby attenuating its progression [87]. Arthritis is a joint disorder resulting in joint pain, it is usually caused by inflammatory substances and joint wear. Studies have found that flaxseed oil was effective in the treatment of knee osteoarthritis, especially in the aspects of ameliorating the severe symptoms and functional status, due of its anti-inflammatory ability [88]. Atefeh Akrami et al. observed a beneficial effect of flaxseed oil consumption on reducing the inflammatory state (including serum IL-6 concentrations) in patients with metabolic syndrome, a chronic disease with an inflammatory and hypercoagulable state [89]. The study showed that compared to olive oil and safflower oil, perilla seed oil in particular has a better protective effect against inflammation of the colon caused by a high-fat diet due to its richness in ω-3 PUFAs, especially ALA, the precursor of EPA and DHA. The mechanism of this feature is that ALA exerts an anti-inflammatory effect by upregulating the expression of peroxisome proliferator-activated receptor *γ* to inhibit the transcription of pro-inflammatory cytokines [85]. Almond oil had significant inhibitory and analgesic effects on an early ear swelling inflammation model, significantly reduced the number of glacial acetic acid-induced torsion occurrences, and increased the pain threshold in hot plate method mice with a more pronounced quantitative effect relationship. Evening primrose oil is extremely high in LA and GLA, which are precursors to the anti-inflammatory eicosanoids, and thus may contribute to the normal functioning of human tissues with anti-inflammatory and anti-proliferative properties [90].

The presence of phenolic compounds, phytosterols, and tocopherols together in VOs may prevent the development of chronic diseases by their anti-inflammatory, antioxidant, neuroprotective, and immunomodulatory activities. Micallef et al. showed that a diet rich in ω-3 polyenoic acids and phytosterols reduced systemic inflammation in hyperlipidemic individuals; in addition, the combination of ω-3 polyenoic acids and phytosterols may result in anti-inflammatory effects through the conversion of ALA (C18:3n-3) to DHA (C22:6n-3), thereby possessing cardioprotective effects [79]. For example, rice bran oil contains tocopherols, tocotrienols, and *γ*- oryzanol, and the results of in vivo and in vitro experiments highlight its ability to ameliorate chronic low-grade inflammation caused by obesity through mediating the expression of inflammation-related factors and macrophage polarization [91]. Grape seed oil may also be considered a suitable candidate for the treatment or prevention of ulcerative colitis because of its anti-inflammatory activity due to the presence of many bioactive components such as phytosterols, vitamin E, and polyphenols [92].

### 3.4. Anti-Obesity

Obesity refers to a certain degree of obvious overweight or a too thick fat layer, especially when caused by TG accumulation [93]. Fat overload in the adipose tissue leads to adipocyte dysfunction, which may stimulate the development of various endocrine and metabolic disorders by affecting glucolipid metabolism and increasing the inflammatory response, and is closely associated with chronic diseases, which include insulin resistance, T2DM, hypertension, dyslipidemia, and CVD [94]. The obesity epidemic is one of the most serious health problems worldwide and obesity is becoming increasingly prevalent in younger people in many countries. VOs with medium and long chain fatty acids are effective in controlling body mass, lowering TG, and improving apolipoprotein metabolism. An understanding of fats and fatty acids in needed to validate the consumption of VOs containing the EFAs in moderation to aid in the control of weight, and better health. High leptin levels and low adiponectin levels are characteristic of obesity. Adiponectin promotes *β*-oxidation by mediating the phosphorylation of adenosine 5‘-monophosphate (5′-AMP) activated protein kinase and activating peroxisome proliferation-activated receptor alpha. Thomas et al. showed that three oils, perilla seed oil, safflower oil and olive oil, may improve high-fat diet-induced obesity by reducing lipogenesis and increasing *β*-oxidation, and these results highlight the importance of replacing saturated fats with unsaturated fats, particularly with PUFA-rich perilla seed oil, which can be seen as a healthy dietary pattern [94]. Akrami et al. clearly showed that consumption of flaxseed oil rich in PUFAs significantly reduced body weight and lowered waist circumference in patients with anti-obesity effects due to a positive linear relationship between eicosanoids in ω-6 PUFA and body mass index (BMI) and waist circumference. The ω-3 PUFA interacted with the regulation of signals related to fat accumulation or energy metabolism [95].

### 3.5. Anti-Cancer

Cancer is a generic term for a large group of diseases that can affect any part of the body. One defining feature of cancer is the rapid creation of abnormal cells that grow beyond their usual boundaries, which can then invade adjoining parts of the body and spread to other organs. According to WHO statistics in 2020, the most common cancers in the world are breast, lung, colon, rectal, and prostate cancers [96].

Phytosterols inhibit cancer cell growth and reduce the metastatic ability of cancer cells through cell cycle arrest and the induction of apoptosis, and have been shown to be effective in breast, colon, leukemia, and prostate cancer metastasis. A daily consumption of phytosterol-rich foods can reduce the risk of cancer by 20% [97]. It is also proposed that the phytosterols exert effects on membrane structure, integrity and fluidity, membrane-bound enzymes, signal transduction pathways, apoptosis, and immune function of host tissues. Yu et al. concluded that rice bran oil containing phytosterols and *γ*-oryzanol may play a vital role in suppressing local inflammation, inhibiting the cancer cell cycle, promoting apoptosis, and enhancing chemo preventive effects, and are considered to be promising adjuvant therapeutic agents for cancer prevention and treatment [98].

In addition, LA helps to inhibit the growth of human breast cancer, colon cancer, skin cancer, stomach cancer, and leukemia [35]. Many studies have shown that treatment of B16-BL6 mouse melanoma and MCF-7 breast cancer cells with LA-rich linseed oil induces apoptosis and disrupts their mitochondrial function in a dose-dependent manner, thereby inhibiting the growth of cancer cell lines in particular. Other studies have shown that flaxseed oil can inhibit the formation and growth of various tumors and cancers, such as colon, breast and skin cancers, and can also enhance the effectiveness of anti-cancer drugs, suggesting further potential for flaxseed oil in anti-cancer therapy [99]. Although many of the substances in flaxseed oil show anti-tumor activity, it is still not possible to clearly elucidate the mechanisms of these components or how they show the ability to reduce the development of cancer [66]. GLA leads to increased levels of polyomavirus enhancer activator 3 protein (Pea3), a transcriptional repressor of human epidermal growth factor receptor 2 (HER-2/neu) in cells, and reduces HE-2/neu promoter activity, thereby reducing the likelihood of breast cancer; it also inhibits the expression of the nm-23 metastasis suppressor gene in cancer cells, thereby inhibiting angiogenesis and cancer cell migration, and achieving the effect of inhibiting the occurrence of metastasis. Thus, evening primrose oil has proven anti-cancer therapeutic functions due to the above mechanisms [90]. Furthermore, perilla seed oil inhibits the development of colon tumors with methyl nitrosourea mainly because the presence of ALA modifies the sensitization of colon cell membranes to carcinogens.

Similar to phytosterols, tocopherols were found to induce programmed cell apoptosis in human colon cancer cell while inhibiting growth of prostate cancer cell [100]. Camellia oil, olive oil, and cottonseed oil blocked intercellular communication and prevented lung metastasis of patients’ melanoma cells [101].

Phenolic compounds found in foods significantly affect their stability, sensory, and nutritional characteristics, and may prevent their spoilage through quenching radical reactions responsible for lipid oxidation [102]. It has also been proven that dietary inclusion of phenolic compounds provides many benefits due to their anti-inflammatory and anti-carcinogenic effects, especially in breast cancer therapy [103]. Canolol is a unique compound in rapeseed oil which has been shown to reduce apoptosis in human cancer SW480 cells [35]. In addition, some studies have shown that the high levels of ellagitannins in pomegranate seed oil promote the repair of biomolecules and cells, and the high levels of lipid peroxidation in punicic acid inhibit the proliferation of cancer cells by affecting the protein kinase C (PKC) pathway, thus showing anti-cancer activity against various forms of cancer, such as breast, prostate, and colorectal cancers [104,105]. 

Currently, squalene is primarily obtained from deep-sea shark liver; however, due to the decreased availability of shark, more attention has been paid to plant sources, such as soybean oils and olive oils, to obtain squalene instead. Olive oil contains 0.2–0.7% squalene, which can regulate the activation of carcinogens, and effectively inhibit the occurrence of colon, lung, and skin tumors induced by rodents [106].

### 3.6. Diabetes Treatment

Diabetes mellitus refers to a type of metabolic disease with the characteristics of high levels of blood glucose [66]. In diabetes, elevated blood glucose levels increase the risk of other complications such as retinopathy, nephropathy, and neuropathy. Clinical evidence has shown that these complications can be controlled well through many dietary therapies. Significant evidence from epidemiological investigations has shown that dietary polyphenols might manage and prevent T2DM. Polyphenols have been reported to show anti-diabetic effects in T2DM patients through increasing glucose metabolism, improving vascular function, and reducing insulin resistance and glycated hemoglobin level. EVOO has been reported to protect against cardiometabolic diseases including CVD, T2DM, and obesity because it contains at least 30 phenolic compounds including simple phenols (tyrosol and hydroxytyrosol), secoroids, and lignans [107]. Daily consumption of polyphenol-rich EVOO might improve metabolic control and the profile of circulating inflammatory adipokines in overweight T2DM patients [108]. In addition, studies have indicated that flaxseed oil plays an important role in the treatment of diabetes by modulating insulin sensitivity in phospholipid membranes, modulating the gut microbiota, or reducing inflammation in the body. Coconut oil has a high total phenolic content and has shown anti-diabetic effect in animal models of type 2 diabetes, thereby ameliorating the adverse effects of diabetes on the liver and kidneys [109]. 

The unsaturated fatty acids in VOs also have an effect on diabetes. Intake of white sesame oil can reduce blood glucose levels, lower oxidative stress, and improve biomarkers of liver and kidney function in patients with T2DM, possibly due to the bioactive components in sesame oil, including MUFA, tocopherols, and phytosterols [110]. Punicic acid (PA) is a PUFA (18:3n-5) belonging to the conjugated linolenic acid isomer, it is particularly abundant in pomegranate seed oil, accounting for about 60.62% to 81.40%. PA exerts its anti-diabetic effect through various mechanisms, such as reduction in inflammatory cytokines, modulation of glucose homeostasis, and its antioxidant properties [111].

### 3.7. Kidney and Liver Protection

The liver functions in metabolism, synthesis, detoxification, and secretion. The main functions of the kidney are filtration, renal tubular reabsorption, and endocrine. Due to the anti-inflammatory capacity of ALA in flaxseed oil, flaxseed oil presents hepatoprotective activity by inhibiting inflammatory signaling pathways. Wang et al. found that flaxseed oil decreases the expression of IL-6, TNF-*α*, and cyclooxygenase-2 (COX-2), leading to the alleviation of lipopolysaccharide-induced liver injury [112]. Kheira et al. highlighted that flaxseed oil improves cisplatin-induced kidney injury through its capability to replace the PUFAs that have been attacked by oxygen FR in the brush-border membrane (BBM) with ω-3 PUFA, which increases the integrity of BBM [113]. 

Furthermore, Omar et al. proved that the administration of flaxseed oil protected against the observed biochemical and histopathological alterations induced by thioacetamide exposure [114]. Therefore, flaxseed oil may rely on its antioxidant effects to protect against thioacetamide-induced renal injury. In addition, sesame oil contains lignans and other acylglycerols which reduce the accumulation of liver fat, inhibit matrix metalloproteinase-2 (MMP-2) and matrix metalloproteinase-9 (MMP-9) by regulating the expression of tissue inhibitor matrix metalloproteinase 1 (TIMP-1), and thus reduce liver damage. Conversely, the approval for this treatment of non-alcoholic steatohepatitis (NASH) has not yet been granted by the US Food and Drug Administration [115]. Only traces of flavonolignans (500 *μ*g/mL) were found in milk thistle seed oil, but its antioxidant, cytoprotective, and hepatoprotective activities have also been reported in vivo and in vitro [116]. 

### 3.8. Other Health Benefits

In addition to the previously mentioned health benefits, VOs contain a wide range of bio-active ingredients, such as lignans in flaxseed oil, nervonic acid in sour cherry seed oil, resveratrol in olive oil, lycopene in tomato seed oil, and DHA (C22:6) in grape seed oil. These special components make significant contributions to their health benefits.

Alzheimer’s disease (AD) is a chronic neurodegenerative disorder. Recently, the supplement of grape seed oil containing DHA (C22:6) to AD rats as medicinal co-treatment or post-treatment caused an obvious augmentation in spatial memory performance and acetylcholine levels [117]. Several epidemiological studies showed a negative correlation between the risk of AD and the intake of DHA. Aside from ALA, flaxseed oil is rich in dietary fibers and lignans that act as anti-oxidants and phytoestrogens, which can alleviate the intensity of symptoms associated with menopause and mental fatigue, as well as benefit autoimmune and neurological disorders [118]. Nervonic acid (C24:1, ω-9), a fatty acid found in high levels in cherry seed oil, is a precursor of neuronal cell membrane glycolipids with a key role in the modulation of ion channels and membrane receptors, and thus acts as a special supplement to promote the regeneration and repair of damaged nerve cells [119]. Lycopene is an acrylic tetraterpenic hydrocarbon with 13 carbon-carbon double bonds, 11 of which are conjugated, which presents potent antioxidant effects. It was reported that tomato seed oil, which is rich in lycopene, effectively inhibited oxidative deterioration, modulated glutathione level, and revived dopamine in the striatum, thus alleviating abnormal behavior of the neurological system [120]. The pharmaceutical functions of polyphenols, resveratrol, and non-flavonoid phenolic compounds was studied in EVOO. Intake of EVOO can effectively prevent crucial neurodegenerative conditions (aging and alcohol-related brain disorders) and neuromuscular disorders [121]. The beneficial effects of epigallocatechin gallate (EGCG), epicatechin, resveratrol, and quercetin supplements were reported in clinical trials; as an example, quercetin supplements are now commercially available in Europe and the U.S. with many health-promotion functions [32,122]. 

Three important factors for the nutritional evaluation of edible oils were introduced by the World Health Organization (WHO): presence of antioxidants; ratio of SFAs, MUFAs, PUFAs, and EFAs content. They suggest a ratio of 1:1.5:1 for SFAs: MUFAs: PUFAs, and a ratio of 0.8–1.0 for total polyunsaturated fatty acid (TPUFA) to total saturated fatty acid (TSFA) [12]. FAs’ ratios to several representative VOs were calculated and are summarized in Table 7. The recommended ratio of SFA: MFA: PUFA is reported for rice bran oil (1:1.8:1.4), custard-apple seed oil (1:2.2:1.1), pumpkin seed oil (1:1.0:1.9), cress oil (1:2.2:2.7), sesame oil (1:2.5:2.4), and corn oil (1:2.0:3.0). Jackfruit seed oil (0.95), olive oil (0.89), custard-apple seed oil (1.08), camellia oil (0.59), avocado seed oil (1.17) and rice bran oil (1.38) show ratios of TPUFA/TSFA closer to the WHO recommended values, while the ratios of pomegranate seed oil (16.42) and evening primrose oil (10.30) were much higher than the recommended values.

The abovementioned health benefits of VOs are relevant to all aspects of people’s lives, and the mechanisms of nutritional ingredients need to be further explored. As for their physiological activity, studies combining lipidomics, proteomics, metabolomics, and nutrigenomics may provide further insights into the mechanisms of the health benefits induced by VOs.

## 4. Conclusions

**Figure 1:**
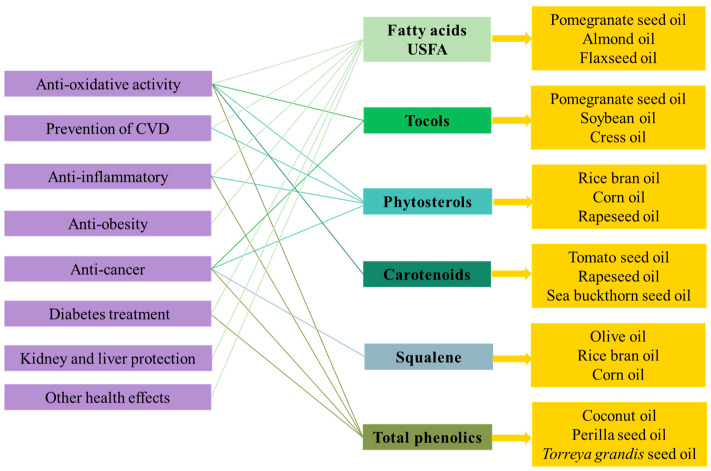
Key components of representative vegetable oils corresponding to their health benefits.

This study offers a comprehensive analysis of 39 VOs, and also covers their chemical compositions, nutritional properties, and health-promoting benefits. VOs are important as part of a healthy dietary pattern since they provide EFAs (Dietary Guidelines for Americans 2020–2025). Strategies to shift the intake towards VOs instead of fats, including butter, shortening and lard, would benefit human health. Adults need an appropriate amount of oil intake of 22–44 g/day depending on the number of calories required per day. The intake of VOs from various sources has distinctive effects on human health including antioxidant activity, anti-cancer, anti-inflammatory, prevention of CVD, anti-obesity, diabetes treatment, kidney and liver protection, and other health effects. These health benefits are ascribed to fatty acids, tocols, phytosterols, carotenoids, squalene, and phenolics content accordingly. The increasing attention given to the functional compounds in VOs and their correlations to human health effects in recent years is due to the increasing public awareness. This review reports that different types of VOs have their own specific advantages and functional nutritional properties; thus, appropriate VOs need to be selected to meet individual needs accordingly. To clarify the functional substances correlation to the health benefits, based on the richness of each key component, the representative VOs are summarized and recommended in Figure 1. 

As shown in the graphical illustration conclusion in Figure 1, FAs, tocols, phytosterols, phenolics, carotenoids, and PUFAs are crucial functional substances exhibiting anti-oxidative ability. For the prevention of CVD, FAs and phytosterols would be benefit; FAs, phytosterols, and total phenolics effect the anti-inflammatory ability; FAs would be the only functional component effective in anti-obesity; tocols, phytosterols, squalene, and total phenolics exhibit anti-cancer ability; FAs and total phenolics exhibit anti-diabetic effects; and FAs could benefit the kidney and liver, as well as neurodegenerative disorders. Therefore, according to Figure 1, in order to prevent cardiovascular diseases, more unsaturated fatty acids and phytosterols should be supplied through the consumption of pomegranate seed oil, almond oil, flaxseed oil, rice bran oil, corn oil, or rapeseed oil. Similarly, considering the prevention of diabetes, more phenolics and FAs need to be incorporated through the intake of coconut oil, perilla seed oil, *Torreya grandis* seed oil, pomegranate seed oil, almond oil, or flaxseed oil. In general, olive oil, rice bran oil, corn oil, and cress oil are recommended as they contain more abundant nutritional ingredients and a closer ratio to the WHO recommended values of SFA: MUFA: PUFA. From another point of view, the long-term intake one kind of vegetable oil has some drawbacks. For example, only consuming olive oil may limit the intake of linolenic acid, and soybean oil contains moderate linolenic acid but contains low anti-oxidative components. Moreover, the current evidence does not favor promoting one vegetable oil over another based on additional health effects, since a variety of different VOs are used in food manufacturing and most people consume a variety of different oils on a daily basis. 

The review summarizes the health benefits of VOs attributed to their characteristic chemical compositions and could provide useful information to consumers on the consumption of VOs in daily diets, especially for patients with diet restrictions. Nevertheless, some of the VOs are in the early stages of research and lack enough reliable data. Compared to the massive consumption of vegetable oils, some minor vegetable oils, such as papaya seed oil, custard-apple seed oil, jackfruit seed oil, and etc., need to be further investigated for their edibility and long-term health effects due to their late development.

## Figures and Tables

**Table 1:** Squalene content of vegetable oils (mg/100 g).

Oils (mg/100 g)	Squalene	References
Olive oil	153.4–747.4	[30]
Rice bran oil	24.0–318.9	[9,18]
Corn oil	6.8–256.8	[31,33]
Pumpkin seed oil	66.7–198.0	[9]
Camellia oil	1.5–159.8	[31,33]
Peanut oil	5.3–132.9	[18,31]
Soybean oil	1.6–92.1	[31,33]
Flaxseed oil	2.4–83.0	[23,32]
Sesame oil	8.8–60.7	[18,32]
Coconut oil	51.5	[32]
Palm oil	6.8–48.3	[31,32]
Perilla seed oil	27.9	[32]
Sunflower oil	5.3–27.1	[31]
Peony seed oil	4.0–18.7	[9,32]
Cottonseed oil	15.8	[32]
Grape seed oil	11.8–13.0	[9,32]
Rapeseed oil	2.1–12.5	[31]
Walnut oil	9.0–12.0	[32]
Safflower oil	5.6	[32]
*Torreya grandis* seed oil	3.0	[9]
Evening Primrose oil	-	[9]

**Table 2:** Carotenoid contents of vegetable oils (mg/kg).

Oils (mg/kg)	*β*-Carotene	Lutein	Zeaxanthin	Lycopene	*α*-Carotene	Total	References
Tomato seed oil	765.7	-	-	-	-	765.7	[9]
Flaxseed oil	34.9–76.9	11.6	1.1	-	-	76.9	[9]
Sea buckthorn seed oil	55.3	-	-	-	-	55.3	[9]
Rapeseed oil	6.0–18.8	32.6–95.0	1.2	-	-	52.6–358.7	[23,35]
Olive oil	36	0.8–4.4	0.8	0.8	3.6	50.3	[35]
Sunflower oil	-	11.6–12.4	-	-	-	3.1–15.3	[22,35]
Camellia oil	21.0	1.6	-	10.0	-	32.6	[35]
Corn oil	0.1	-	-	-	-	0.1	[9]
Rice bran oil	-	-	-	-	-	0	[9]
Peanut oil	-	-	-	-	-	1.8	[35]
Cress oil	4.3	1.0	0.2		-	5.3	[36]

-, Not detected.

**Table 3:** Total phenolic content of vegetable oils (mg/kg).

Oils (mg/kg)	Phenolic Acids	Total Phenolic	References
Coconut oil	4.250	21.0–59,300.0	[40,44]
*Torreya grandis* seed oil	-	12,630.0	[9]
Perilla seed oil	-	38.6–11,090.0	[9]
Rice bran oil	0.004	14.4–10,220.0	[45]
Pomegranate seed oil	-	90.0–9000.0	[13]
Flaxseed oil	2.570	4.0–3073.0	[23,45]
Pumpkin seed oil	164.800	3.9–2360.0	[45]
Olive oil	0.841	23.0–2180.0	[45]
Sunflower oil	0.178	4.8–1920.0	[22,45]
Rapeseed oil	3.710	10.3–1654.5	[23,35,45]
Sesame oil	-	0.4–1337.5	[45]
Avocado seed oil	-	11.6–1301.7	[45]
Grape seed oil	92.490	5.1–1155.0	[45]
Safflower oil	-	26.2–711.0	[45]
Evening Primrose oil	-	48.6–679.0	[9,45]
Almond oil	-	644.5	[9]
Soybean oil	-	4.2–643.7	[45]
Peanut oil	0.016	5.7–501.3	[45]
Palm oil	-	18.2–403.0	[45]
Camellia oil	31.243	19.8–400.0	[9,45]
Cottonseed oil	-	98.6	[46]
Corn oil	8.850	12.6–53.6	[45]
Peony seed oil	-	49.4	[46]
Palm kernel oil	-	3.2–27.2	[45]
Walnut oil	0.022	14.0–26.0	[45,47]

-, Not detected.

**Table 4:** Function of vegetable oils in antioxidant activity.

Functional Components	Oil Types	Mechanisms	References
Tocols	Pomegranate seed oil Soybean oil Cress oil	Primary or chain breaking antioxidants; tocopherols can provide electrons to FR to make them become inactive compounds with the exchange of becoming tocopherol FR, which is easy excrete in feces and urine after metabolism.	[9,52]
Phytosterols	Rice bran oil Corn oil	Possible primary or chain breaking antioxidant.	[52]
Phenolics	Coconut oil *Torreya grandis* seed oil	Primary or chain breaking antioxidants. Secondary or preventive antioxidants act as chelators of metal ions. Stabilize and prevent decomposition of hydroperoxides. Phenolic compounds can prevent the generation of FR in the body and block the oxidation reaction of PUFAs or LDLs induced by FR.	[52]
Carotenoids	Tomato seed oil Sea buckthorn seed oil	Secondary or preventive antioxidants act as singlet oxygen quenchers. Primary or chain breaking antioxidants. Carotenoids are able to donate an electron and neutralize FR, resulting in the suppression of excess FR production to inhibit the deterioration of internal redox balance and terminate some chain reactions.	[52]
Fatty acids	Flaxseed oil	n-3 PUFAs can reduce mitochondrial dysfunction and endothelial cell apoptosis associated with oxidative stress by increasing the activity of endogenous antioxidant enzymes.	[53]

**Table 5:** Function of vegetable oils in preventing cardiovascular diseases.

Functional Components	Oil types	Mechanisms	References
MUFA	Almond oil Olive oil	Oleic acid can decrease plasma triacylglycerol and cholesterol concentrations.	[68]
PUFA	Flaxseed oil Pomegranate seed oil	LA helps to break down cholesterol by promoting cholesterol 7*α*-hydroxylase (CYP7) activity. LA enhances transcription of the liver X receptor (LXR*α*) gene via peroxisome proliferator-activated receptors (PPARs). In turn, LXR*α* upregulates the expression of the CYP7 gene; EPA and DHA protect the blood vessels and heart by regulating membrane phospholipids, improving cardiac mitochondrial function and energy production, and lowering triglyceride concentrations.	[69,70]
Phytosterols	Rice bran oil Corn oil Rapeseed oil	Inhibits the absorption of intestinal cholesterol.	[10]

**Table 6:** Anti-inflammatory function of vegetable oils.

Functional Components	Oil Types	Mechanisms	References
USFA	Flaxseed oil Perilla seed oil Almond oil	ALA exerts an anti-inflammatory effect by upregulating the expression of Peroxisome proliferator-activated receptor *γ* to inhibit the transcription of pro-inflammatory cytokines. EPA and DHA reduce the production of AA-derived eicosanoids by competing with AA for incorporation into cell membrane phospholipids, reduce AA release from the membrane, inhibit the action of the enzymes COX-2 and 5-lipoxygenase (5-LOX) on AA, or compete with AA for metabolism by COX and LOX enzymes.	[69,85]
Phytosterols	Rice bran oil	Phytosterols can suppress the transcription of inflammatory genes in macrophages.	[86]

**Table 7:** Proportion of fatty acids in vegetable oils.

FA	SFA	MUFA	PUFA	SFA: MUFA: PUFA	TPUFA/TSFA
Soybean oil	16.18	23.88	60.98	1:1.5:3.8	3.77
Rapeseed oil	7.52	72.77	29.36	1:9.7:3.9	3.90
Palm oil	44.15	46.30	9.38	4.7:4.9:1	0.21
Peanut oil	10.70	71.10	18.20	1:6.6:1.7	1.70
Sunflower oil	11.54	28.30	67.75	1:2.5:5.9	5.87
Cottonseed oil	7.11	14.43	40.23	1:2.0:5.7	5.66
Corn oil	16.60	33.67	49.74	1:2.0:3.0	3.00
Camellia oil	17.26	72.50	10.10	1.7:7.2:1	0.59
Coconut oil	92.10	6.20	1.60	57.6:3.9:1	0.02
Olive oil	20.19	76.62	18.00	1.1:4.3:1	0.89
Flaxseed oil	12.90	23.00	76.94	1:1.8:6.0	5.96
Jackfruit seed oil	49.13	4.15	46.72	11.8:1:11.3	0.95
Papaya seed oil	19.92	76.10	3.96	5.0:19.2:1	0.20
Avocado seed oil	11.74	73.71	13.78	1:6.3:1.2	1.17
Pomegranate seed oil	5.35	6.79	87.87	1:1.3:16.4	16.42
Cheery oil	12.80	39.60	46.30	1:3.1:3.6	3.62
Sweet cherry seed oil	12.20	39.49	44.32	1:3.2:3.6	3.63
Sour cherry seed oil	7.46	38.49	54.05	1:5.2:7.2	7.25
Custard-apple seed oil	23.04	51.04	24.96	1:2.2:1.1	1.08
Cress oil	16.90	37.30	45.80	1:2.2:2.7	2.71
Pumpkin seed oil	25.20	25.54	48.14	1:1.0:1.9	1.91
Sesame oil	16.90	42.00	41.20	1:2.5:2.41	2.44
Rice bran oil	23.63	43.71	32.66	1:1.8:1.4	1.38
Almond oil	8.35	77.07	22.59	1:9.2:2.7	2.71
Evening Primrose oil	8.10	9.40	83.40	1:1.2:10.3	10.30
Perilla seed oil	8.22	12.89	76.25	1:1.6:9.3	9.28
Milk thistle seed oil	15.02	35.94	48.81	1:2.4:3.2	3.25
Tomato seed oil	24.48	21.79	53.70	1.1:1:2.5	2.19

## Data Availability

Not applicable.

## Appendix A

**Table A1:** Fatty acid composition of vegetable oils (%).

Oils	Lauric C12:0	Myristic C14:0	Pentadecanoic C15:0	Palmitic C16:0	Palmitoleic C16:1	Margaric C17:0	Stearic C18:0	Oleic C18:1n-9	Linoleic C18:2n-6	Linolenic C18:3n-3	Arachidic C20:0	Gadoleic C20:1	Behenic C22:0	Lignoceric C24:0	Others	SFA	MUFA	PUFA	References
Soybean oil	-	0.12	-	12.20	0.16	0.04	6.50	29.10	53.33	7.65	0.32	0.22	0.27	0.13	-	16.18	23.88	60.98	[15]
Rapeseed oil	-	0.04	0.02	4.60	0.23	0.07	1.70	63.44	20.65	8.71	0.87	9.10	0.27	0.04	-	7.52	72.77	29.36	[15]
Palm oil	-	0.88	0.03	38.08	0.14	-	4.50	45.10	9.01	0.14	0.39	0.17	0.06	0.07	0.89	44.15	46.30	9.38	[24]
Peanut oil	-	0.04	-	7.50	0.07	0.07	2.10	71.10	18.20	0.12	1.01	-	-	-	-	10.70	71.10	18.20	[16]
Sunflower oil	-	0.09	0.02	6.35	0.12	0.02	3.92	28.00	67.58	0.17	0.22	0.18	0.66	0.26	-	11.54	28.30	67.75	[15]
Cottonseed oil	-	0.80	-	24.20	0.70	-	2.30	17.40	53.20	0.20	0.20	0.10	0.10	0.10	-	27.80	18.20	53.40	[17]
Corn oil	-	-	-	12.94	1.70	-	2.12	31.97	48.97	0.76	0.68	-	0.39	0.47	-	16.60	33.67	49.74	[12]
Camellia oil	-	-	-	13.80	0.06	-	3.10	72.50	9.50	0.60	0.36	-	-	-	-	17.26	72.50	10.10	[9]
Coconut oil	47.70	19.90	-	-	-	-	2.70	6.20	1.60	-	-	-	-	-	13.62	92.10	6.20	1.60	[16]
Olive oil	-	0.08	-	16.50	1.80	0.07	2.79	74.52	16.40	1.60	0.49	0.30	0.16	0.17	-	20.19	76.62	18.00	[15]
Flaxseed oil	-	0.05	0.01	6.42	0.20	0.04	5.96	22.56	15.88	61.06	0.20	0.21	0.14	0.13	0.12	12.90	23.00	76.94	[15]
Jackfruit seed oil	-	-	-	36.20	-	-	3.54	4.15	35.11	2.82	-	-	-	-		49.13	4.15	46.72	[123]
Papaya seed oil	-	0.21	-	13.70	0.11	-	5.63	75.89	3.62	0.34	0.38	0.10	-	-		19.92	76.10	3.96	[14]
Avocado seed oil	-	-	-	11.62	5.91	-	0.12	67.80	13.67	0.11	-	-	-	-		11.74	73.71	13.78	[124]
Pomegranate seed oil	-	-	-	2.41	-	-	2.42	6.18	5.54	46.27	0.52	-	-	-	36.66	5.35	6.79	87.87	[13]
Cheery oil	-	-	-	7.60	0.20	-	5.20	39.40	46.20	0.10	-	-	-	-		12.80	39.60	46.30	[125]
Sweet cherry seed oil	0.13	-	-	9.05	0.47	-	3.02	35.05	41.45	-	-	-	-	-		12.20	39.49	44.32	[126]
Sour cherry seed oil	-	0.02	-	4.92	0.29	0.05	1.60	37.89	42.42	0.11	0.64	0.31	0.15	0.08		7.46	38.49	54.05	[127]
Custard-apple seed oil	-	-	-	14.86	-	-	8.18	51.04	23.06	1.90	-	-	-	-		23.04	51.04	24.96	[128]
Cress oil	-	0.13	-	10.13	0.20	-	2.84	22.45	12.61	36.25	2.06	9.88	0.33	0.02		15.51	35.06	48.86	[129]
Mustard oil	-	-	-	2.19	0.17	-	1.17	10.16	15.58	11.70	0.98	5.48	1.40	1.18		5.73	66.98	27.28	[12]
Walnut oil	-	0.03	-	7.35	0.15	0.10	3.00	17.20	60.30	12.00	0.04	0.20	0.03	0.02		10.57	17.55	72.30	[130]
Wheat germ oil	0.07	-	0.04	17.40	0.21	0.03	0.70	12.70	59.70	1.20	7.91	-	-	-	-	18.20	20.90	61.00	[16]
Safflower oil	-	0.10	-	6.70	0.08	0.04	2.40	11.5	79.00	0.15	0.43	0.13	0.75	0.12	-	9.30	11.60	79.10	[16]
Grape seed oil	-	-	-	7.20	-	-	4.80	19.90	68.10	0.10	-	-	-	-		12.00	19.90	68.20	[131]
Pumpkin seed oil	-	-	-	17.58	-	-	7.62	25.54	47.45	0.69	-	-	-	-		25.20	25.54	48.14	[126]
Sesame oil	-	-	-	9.70	0.11	-	6.50	41.50	40.90	0.21	0.63	0.40	0.14	0.32		16.90	42.00	41.20	[16]
Rice bran oil	-	0.35	-	19.34	0.29	-	2.00	43.42	32.04	0.59	1.09	-	0.33	0.51		23.63	43.71	32.66	[12]
Almond oil	-	-	-	6.58	0.48	-	1.64	76.59	21.86	0.73	0.13	-	-	-		8.35	77.07	22.59	[132]
Date Palm Seed Oil	17.26	10.74	0.04	9.88	0.06	0.05	2.82	48.67	8.13	0.03	0.37	0.44	0.27	0.14	1.09	42.22	49.59	8.16	[24]
Evening Primrose oil	-		-	6.00	0.10		1.60	8.30	73.80	0.10	0.30	0.30	0.10	-	10.30	8.10	9.40	83.40	[17]
Perilla seed oil	-	0.02	-	6.33	0.12	0.03	1.70	12.61	18.30	59.87	0.11	0.13	0.02	0.01		8.22	12.89	76.25	[133]
*Eucommia ulmoides Oliver* seed oil	-	0.09	-	9.82	0.3	0.11	2.59	16.86	12.66	56.79	0.3	0.18	0.19	0.11	-	13.21	17.34	69.45	[9]
Peony seed oil	-	-	-	5.80	-	-	1.97	21.76	24.60	44.38	-	-	-	-		8.17	22.37	69.46	[134]
Sea buckthorn seed oil	-	0.11	-	8.73	0.61	-	2.58	22.33	31.6	28.24	0.45	-	-	-	-	11.87	22.94	59.84	[9]
*Acer truncatum Bunge* seed oil	-	-	-	4.19	0.18	-	2.4	25.8	37.35	1.85	0.25	8.23	0.78	0.32	18.60	7.94	52.81	39.20	[9]
*Torreya grandis* seed oil	-	-	-	9.18	-	0.07	2.35	22.89	45.39	0.81	0.14	1.54	-	-		11.74	24.43	63.44	[135]
Milk thistle seed oil	-	0.07	-	6.25	18.98	0.08	4.03	15.78	48.70	0.11	2.33	0.72	1.86	0.40	-	15.02	35.94	48.81	[136]
Tomato seed oil	-	-	-	17.14	-	-	5.21	21.79	53.7	-	2.13	-	-	-	-	24.48	21.79	53.70	[9]

Values are expressed as a relative percentage of total fatty acids. -, not detected; SFA, total saturated fatty acids; UFA, unsaturated fatty acids; MUFA, total monounsaturated fatty acids; PUFA, total polyunsaturated fatty acid; UFA, total unsaturated fatty acids.

**Table A2:** Tocols contents of vegetable oils (mg/kg).

Oils (mg/kg)	*α*-Tocopherol	*β*-Tocopherol	*γ*-Tocopherol	*δ*-Tocopherol	*α*-Tocotrienol	*β*-Tocotrienol	*γ*-Tocotrienol	*δ*-Tocotrienol	Total	*α*-TE (mg)	References
Soybean oil	101.6	9.1	1639.7	304.7	-	-	-	-	1774.6	181.2	[20,22]
Rapeseed oil	267.0	-	398.0	23.0	-	-	-	-	718.0	235.4	[35]
Palm oil	151.2	4.8	19.6	-	203.6	-	303.2	163.9	843.6	297.0	[24]
Palm kernel oil	13.0	-	-	-	21.0	-	-	-	34.0	19.3	[22]
Peanut oil	36.9	21.8	188.0	13.0	-	-	-	-	259.6	178.9	[20]
Sunflower oil	570.5	95.8	56.2	-	-	-	-	-	722.5	645.9	[20]
Cottonseed oil	386.2	7.8	380.7	2.3	-	-	-	-	777.0	428.1	[22]
Corn oil	317.1	39.9	518.7	28.6	-	-	-	-	886.5	-	[9,137]
Rice bran oil	299.5	14.7	73.6	10.0	518.1	-	823.4	48.9	1556.8	-	[9,20]
Coconut oil	4.0	-	3.0	3.0	8.1	1.0	14.3	2.0	35.0	5.9	[20,22]
Olive oil	290.0	10.0	10.0	1.0	-	-	-	-	311.0	142.5	[22,138]
Date Palm Seed Oil	124.0	16.8	167.8	5.3	213.6	-	134.9	44.8	707.5	-	[24]
Sesame oil	6.1	27.1	724.1	10.4	-	-	13.5	-	775.0	52.5	[20,22]
Camellia oil	378.0	-	26.0	12.0	-	-	-	-	416.0	-	[18]
Perilla seed oil	23.0	2.0	831.0	14.0	-	-	-	-	870.0	-	[133]
Safflower oil	451.2	7.0	32.9	7.0	-	-	-	-	498.0	-	[22]
Grape seed oil	67.0	2.1	7.3	0.24	521.1	7.7	132.2	18.3	755.8	-	[139]
Peony seed oil	12.1	-	484.2	25.6	-	-	-	-	521.9	-	[9]
*Torreya grandi*s seed oil	30.0	510.0	30.0	-	-	-	-	-	570.0	-	[9]
Walnut oil	13.9	-	169.2	26.3	-	-	-	-	209.4	-	[9]
Sea buckthorn seed oil	325.0	62.7	459.1	51.3	-	-	-	-	898.1	-	[9]
Evening Primrose oil	118.6	-	273.3	-	-	-	-	-	391.9	-	[9]
Almond oil	179.6	21.9	8.0	-	0.9	-	-	-	210.4	-	[132]
Cress oil	80.0	60.0	1550.0	190.0	-	-	-	-	1754.0	-	[138]
*Eucommia ulmoides Oliver* seed oil	8.2	6.3	906.2	146.9	-	-	-	-	1067.6	-	[9]
Flaxseed oil	21.1	1.7	407.0	14.6	-	-	-	-	553.0	44.7	[9,22]
Pomegranate seed oil	139.0	-	3829.0	96.0	-	-	-	-	5246.0	-	[19]
Milk thistle seed oil	286.2	3.6	14.2	14.2	-	-	-	-	318.3	-	[136]
Tomato seed oil	23.4	49.0	136.9	136.5	-	-	-	-	345.8	-	[9]

-, Not detected.

**Table A3:** Phytosterol contents of vegetable oils (mg/100 g).

Oils (mg/100 g)	Brassicasterol	Ergosterol	Campesterol	Campestanol	Stigmasterol	*β*-Sitosterol	∆5-Avenasterol	Δ7-stigmastenol	Cycloartanol	Cycloartenol	Methylene-Cycloartanol	Sitostanol	Total	References
Soybean oil	12.80	1.98	62.68	5.91	87.28	360.03	7.21	5.10	2.72	4.71	4.34	-	448.30	[20,28]
Rapeseed oil	136.64	2.54	267.50	2.83	25.67	394.11	40.92	18.46	1.10	17.26	5.28	-	893.84	[28]
palm oil	-	-	18.89	-	6.91	55.58	1.83	-	-	-	-	-	83.20	[22]
Palm kernel oil	-	-	12.05	-	14.02	91.96	7.99	1.05	-	-	-	-	131.00	[22]
Peanut oil	-	1.56	41.19	4.95	48.16	189.12	19.70	-	0.73	9.42	4.92	-	319.75	[28]
Sunflower oil	0.58	0.25	28.36	1.71	18.69	324.00	12.45	63.88	0.18	8.85	11.27	18.95	453.30	[20,28]
Cottonseed oil	-	-	34.01	-	5.20	412.79	19.84	-	-	-	-	-	472.30	[22]
Corn oil	4.13	0.87	197.32	74.53	45.53	661.70	97.92	-	1.89	15.85	12.97	112.44	1032.07	[28,29]
Camellia oil	-	2.62	16.52	0.22	22.11	50.09	1.81	-	29.11	17.84	2.33	116.97	142.64	[28]
Coconut oil	-	-	11.41	-	13.07	51.58	16.28	-	-	-	-	-	97.50	[22]
Olive oil	-	0.83	25.85	1.61	21.13	152.05	29.73	-	2.79	19.44	34.58	-	288.02	[28]
Flaxseed oil	1.66	-	175.9	4.17	53.50	237.5	56.01	-	0.99	78.67	39.29	29.59	775.20	[9,28]
Sesame oil	-	2.05	90.30	7.48	86.89	555.0	98.79	5.98	0.83	23.79	4.75	-	637.60	[20,28]
Pumpkin seed oil	-	-	2.30	-	1.30	182.30	-	-	-	-	-	-	185.90	[140]
Perilla seed oil	-	-	18.70	-	10.50	318.60	-	-	-	-	-	-	347.80	[9]
Safflower oil	-	-	50.0	3.39	25.40	168.30	9.07	73.01	-	-	-	23.29	412.50	[9,22]
Grape seed oil	-	-	30.40	-	47.92	230.64	10.33	-	-	-	-	19.53	338.83	[139]
Peony seed oil	1.77	2.65	21.32	4.25	2.57	258.71	3.64	-	-	6.37	65.90	-	367.30	[28]
*Torreya grandis* seed oil	-	-	20.00	-	5.90	109.30	-	-	-	-	-	-	135.20	[141]
Walnut oil	2.14	0.67	31.53	6.94	32.80	165.23	5.99	-	0.44	15.33	10.97	-	272.04	[28]
Sea buckthorn seed oil	-	-	-	-	-	104.70	-	-	-	-	-	-	104.70	[9]
Evening Primrose oil	-	-	18.20	-	29.2	44.00	-	-	-	-	-	-	91.40	[9]
Almond oil	-	-	22.61	-	19.67	171.68	20.58		-	-	-	-	258.12	[132]
Rice bran oil	6.33	2.17	226.43	221.20	132.90	735.17	157.41	-	31.08	156.25	222.88	-	1891.82	[28]
Sour cherry seed oil	-	-	18.70	0.40	1.00	601.83	17.74	-	-	-	-	18.50	678.54	[127]
Milk thistle seed oil	-	-	24.27	2.75	30.07	166.80	15.42	-	-	8.50	12.21	-	508.85	[136]
Pomegranate seed oil	-	-	36.30	-	16.30	354.20	-	-	-	-	-	23.60	552.70	[19]

-, Not detected.
